# Datasets on mathematical modeling of multi-product multi-stage production to analyze the relationship between production yield, demand, and costs

**DOI:** 10.1016/j.dib.2019.01.028

**Published:** 2019-01-18

**Authors:** Işılay Talay, Öznur Özdemir-Akyıldırım

**Affiliations:** a School of Business and Social Sciences, Antalya Bilim University, Çıplaklı District, Akdeniz Boulevard, No:290 A, Döşemealtı, Antalya, Turkey; b Faculty of Economics and Administrative Sciences, Akdeniz University, Dumlupınar Boulevard, Campus, Antalya, Turkey

## Abstract

The data presented in this article are related to the research article “Optimal procurement and production planning for multi-product multi-stage production under yield uncertainty” (Talay and Özdemir-Akyıldırım, in press) [1]. The data includes: 1) the input parameters (production yield, demand, and costs) collected through comprehensive review of the literature and diversified further to enrich the analytical results, and 2) results from mathematical modeling and analysis on the optimal procurement and semi-processed material allocation decisions for different parameter sets. The dataset is particularly constructed for a production system with two stages and three final products.

**Specifications table**

**Table:** 

Subject area	*Business*
More specific subject area	*Operations Management*
Type of data	*Excel spreadsheets*
How data was acquired	*Collected through comprehensive review of the literature as described in**[1]**and further diversified*
Data format	*Raw and analyzed*
Experimental factors	*Input data collected for cost parameters were filtered based on the relative comparison between costs for different end products rather than the absolute values of the costs*
Experimental features	*Input data is fed into the mathematical model in**[1]**and the analysis results were also obtained through the algorithms in**[1]*
Data source location	Antalya Bilim University, School of Business and Social Sciences, Antalya, Turkey; Akdeniz University, Faculty of Economics and Administrative Sciences, Antalya, Turkey
Data accessibility	*Data is with this article*
Related research article	“Optimal procurement and production planning for multi-product multi-stage production under yield uncertainty” (Talay and Özdemir-Akyıldırım, in press) [1]

**Value of the Data**

• The input data, in other words the parameter values, have been collected through a comprehensive review of the literature on production yield. The literature included data on mostly single-product models, there were very few multi-product oriented models. Therefore, the data collected have been further diversified and extended to be applied to a multi-product model. Thus, datasets in this article could be exported to be used as input for other multi-product oriented production yield models in the future.
• The input data could be further extended to include other types of parameters representing different production environment dynamics, for instance production lead times, to provide input for more complex models and numerical studies.
• The output data obtained from the analysis of the mathematical model in [1] could be exported to compare the results after the input data is applied to different models.

## 1 Data

The input data collected from [2], [3], [4] was further diversified and analyzed through the mathematical model in [1]. First, the parameter values previously used in the literature was collected from the sources cited above; then, these parameters were further extended to be made applicable for the model in [1]; finally different datasets were formed through combinations of various parameter values. The three datasets formed are titled as “Dataset j on mathematical modeling of multi-product multi-stage production.xlsx” with *j* = 1, 2, and 3. The datasets contain the range of values for the parameters described in the table below and provide the optimal values for the decision variables (*X*_i_ with *i* = 0, 1, 2, and 3) along with the optimal value of the objective function (*V*_1_ (*X*_0_)) as described in the next section.

The table below presents the descriptions of the parameters and all the values used for each parameter to form the combinations at the datasets (Table 1).

**Table 1:** Parameter values for the input data.

**Parameters (*i* = 1, 2, 3)**	**Description**	**Values included in the datasets**
*P*_i_	price per unit for product i	*P*_1_ = 40,50; *P*_2_ = 50; *P*_3_ = 50
*s*_i_	salvage value per unit for product i	*s*_i_ = 0.2*P_i_
*c*^p^	procurement and first stage production cost per unit	*c*^p^ = 10, 20, 30
*c*_i_^pe^	penalty cost for unmet demand per unit of product i	*c*_i_^pe^ = 10, 20
*c*_i_°	excessive production cost per unit for product i	*c*_i_^o^ = - *s*_i_
*c*_i_^u^	insufficient production (demanded but not satisfied) cost per unit for product i	*c*_i_^u^ = *P*_i_ + *c*_i_^pe^
*c*_i_	production cost per unit for the second stage for product i	*c*_i_ = 10, 20, 30
*p*_0_	production yield (probability of producing a usable product) at the first production stage for product	*p*_0_ = 0.3, 0.5–1 (with increments of 0.05)
*p*_i_	production yield (probability of producing a usable product) at the second production stage for product i	*p*_1_ = 0.3, 0.5–1 (with increments of 0.05), *p*_2_ = *p*_3_ = 0.3, 0.7, 1.0
*D*_i_	demand for product type i	*D*_i_ = 10, 20, 30, 40, 50
*θ*	discount factor	*θ* = 1

The output data are the solutions obtained from the model in the form of optimal decision variables and objective function values. The specifics of the model are discussed in the next section.

## 2 Experimental design, materials, and methods

The input data were generated through the search of the literature as presented in the above table. The ranges of the parameters above were used to generate a dataset for the parameter values, and the model described briefly below and in detail in Section 2 of [1] was solved via the algorithms 1 and 2 in Section 3 of [1]. The algorithms were coded via Matlab software to provide the optimal values of the decision variables and the objective function value as described below. Thus, the datasets were formed through solving the two-stage stochastic optimization model below via the algorithms in [1] using the Matlab software. The forms of the output data are the decision variables and the objective function values. The data are presented in the files included with this data article.

1. *Decision variables*:
  *X*_0_: procurement amount to start the production at the first stage
  *X*_i_: semi-processed item (output of the first stage) amount allocated to product i for second stage
2. Constraint parameter
  *Y*: amount of usable semi-processed items that survived the first stage, *Y* ~ Binomial (*X*_0_, *p*_0_)
3. *Objective function values*
  *V*_1_ (*X*_0_): objective function value depending on the choice of the decision variable X_0_.

First-stage of the production model for the multi-product multi-stage production problem (${Ζ}^{*}$denotes the set of nonnegative integers)

$${V_{1}\left(X_{0}\right)=\min}_{X_{0}}X_{0}c^{p}+\theta \sum_{y=0}^{X^{0}}P(Y=y)V_{2}(Y=y)$$

$$s.t.X_{0}\geq \sum_{i}D_{i}$$

$$X_{0}\in {Ζ}^{*}$$

Second-stage of the production model for the multi-product multi-stage production problem

$$\min_{\begin{array}{c} X_{i}\\ i=1,\ldots ,n \end{array}}{\sum_{i=1}^{n}E\left[{-D}_{i}P_{i}+X_{i}c_{i}+\max\left(0,\left(B\left(X_{i},p_{i}\right)-D_{i}\right)\right)c_{i}^{o}+\max\left(0,\left(D_{i}-B\left(X_{i},p_{i}\right)\right)\right)c_{i}^{u}\right]X}_{0}c^{p}$$

$$s.t.\sum_{i=1}^{n}X_{i}=Y$$

$$X_{i}\in {Ζ}^{*}$$

The datasets titled as “Dataset 1 on mathematical modeling of multi-product multi-stage production.xlsx” present how the optimal *X*_0_ value changes when the p_0_ values shift from 0.5 to 1.0 in increments of 0.05; whereas the dataset titled as “Dataset 2 on mathematical modeling of multi-product multi-stage production.xlsx” present how the optimal X_0_ value changes with the changes in the *p*_i_ values. Finally, the dataset titled as “Dataset 3 on mathematical modeling of multi-product multi-stage production.xlsx” present how the optimal *X*_i_ values change with the changes in the *p*_i_ values.
