# Supplementary material for: Datasets on mathematical modeling of multi-product multi-stage production to analyze the relationship between production yield, demand, and costs
Source: Data Brief. 2019 Jan 18;22:1027–30. doi: 10.1016/j.dib.2019.01.028 (PMC6355965; doi:10.1016/j.dib.2019.01.028)
Supplement: Supplementary file 1 — Supplementary material [file mmc1.docx]

**Conflict of Interest Form**

There are no conflict of interest.
